# Gender modified association of oral health indicators with oral health-related quality of life among Korean elders

**DOI:** 10.1186/s12903-022-02104-6

**Published:** 2022-05-06

**Authors:** Huong Vu, Phuc Thi-Duy Vo, Hyun-Duck Kim

**Affiliations:** 1 Department of Preventive and Social Dentistry, School of Dentistry, Seoul National University, 101 Daehak-Ro, Jongno-Gu, Seoul, 03080 Republic of Korea; 2 Department of Immunology and Molecular Microbiology, School of Dentistry and Dental Research Institute, Seoul National University, Seoul, Republic of Korea; 3 Dental Research Institute, Seoul National University, Seoul, Republic of Korea

**Keywords:** Oral health indicator, OHRQoL, OHIP, Gender

## Abstract

**Objective:**

To evaluate the association between oral health-related quality of life (OHRQoL) and oral health indicators including dental status, total occlusion force (TOF), number of natural and rehabilitated teeth (NRT), number of natural teeth (NT), and to explore the effect modification on the association by gender among Korean elders.

**Methods:**

A total of 675 participants aged 65 or above recruited by a cluster-based stratified random sampling were included in this cross-sectional study. The 14-items Korean version of the Oral Health Impact Profile (OHIP) was used to measure OHRQoL. The responses about OHIP were dichotomized by the cut-off point of 'fairly often' to determine the ‘poor’ versus ‘fair’ OHRQoL. Age, gender, education level, alcohol drinking, smoking, metabolic syndrome, frailty, and periodontitis were considered as confounders. Multiple multivariable logistic regression analyses were applied to assess the adjusted association between oral health indicators and OHRQoL. Gender stratified analysis was also applied to explore the effect modification of the association.

**Results:**

The prevalence of poor OHRQoL was 43.0%, which was higher in women, less-educated elders, alcohol non-drinkers and frailty elders (*p* < 0.05). Elders with poor OHRQoL also showed lower values of oral health indicators than elders with fair OHRQoL (*p* < 0.05). Those with NRT ≤ 24, NT ≤ 14, and TOF < 330 N increased the risk of poor OHRQoL by 2.3 times (OR = 2.26, confidence interval [CI] 1.54–3.31), 1.5 times (OR = 1.45, CI 1.02–2.07), and 1.5 times (OR = 1.47, CI 1.06–2.04), respectively. In women, the association of NRT ≤ 24 with poor OHRQoL increased from OR of 2.3 to OR of 2.4, while, in men, the association of TOF < 330 N with poor OHRQoL increased from OR of 1.5 to OR of 3.2.

**Conclusion:**

Oral health indicators consisting of TOF, NRT, and NT were independently associated with poor OHRQoL among Korean elders. Gender modified the association of TOF and NRT. Preventive and/or curative management for keeping natural teeth and the rehabilitation of missing teeth to recover the occlusal force may be essential for reducing poor OHRQoL.

**Supplementary Information:**

The online version contains supplementary material available at 10.1186/s12903-022-02104-6.

## Introduction

According to the United Nations, population aging is considered as one of global demographic megatrends; by 2050, the proportion of elderly will reach 20% of the population [1]. This brings a heavy burden of systemic diseases and poor oral condition in elders to the health care system [2, 3]. Therefore, health promotion and disease prevention programs are expected to increase to deal with health issues in the geriatric population [4]. Because the oral health is associated with general health [5], it is crucial to assess oral health problems in the elderly from the standpoint of public oral health. Overall oral health status could be evaluated through Oral health-related quality of life (OHRQoL), which is an integral part of general health and well-being that focuses on oral health [6]. Since it has had important clinical and epidemiological implications, it has gained increasing attention in dentistry, especially in the fields of public oral health among the elders.

Various tools for evaluating the OHRQoL have been developed. Among them, the Oral Health Impact Profile (OHIP), developed by Slade and Spencer in 1994 [7], was one of the most comprehensive assessments for measuring the OHRQoL. The OHIP has 49 items in seven domains: functional limitation, pain, psychological discomfort, physical disability, psychological disability, social disability, and handicap. Later on, a shortened OHIP-14 was derived from the OHIP-49 by using two items for each domain [8]. Korean version of the OHIP and its short-form OHIP-14K were developed in 2007 [9], and has been applied in various geriatric researches for Koreans [10, 11].

Notwithstanding some limitations of previous studies, the impact of oral health indicators on quality of life (QoL) and OHRQoL has been widely investigated. A lower number of remaining teeth was associated with poor QoL [12–15]. The impairment of chewing ability also had a negative impact on OHRQoL in both middle aged adults and elders [11, 12]. Furthermore, elders with denture were more likely to have a poor OHRQoL than those with dentate [15, 16]. However, most studies have not considered sufficient covariates including socio-demographic factors, health behaviors, and general/oral health problems. Moreover, no study has investigated the comparison of association between the oral health indicators encompassing dental status, TOF, NRT, and NT with OHRQoL simultaneously.

Potential confounders could be listed on the association of oral health indicators with OHRQoL. Younger elders [15, 17], females [17, 18], and lower education [19, 20] were associated with poor OHRQoL. Smoking [10], alcohol consumption [10, 20] also had a negative impact on OHRQoL. Moreover, many studies indicated that general health problems (metabolic syndrome [21, 22], frailty [23]), and periodontitis [24] were associated with poor QoL. Interestingly, women were more likely to have poor OHRQoL than men [17, 18]. However, the effect modification by gender on the association of oral health indicators and OHRQoL has not been explored. Thus, more clarified evidence is needed form well designed studies in different methods and populations to established the association between two.

On the basis of this collective infomation, we made two hypotheses. First, oral health indicators including dental status, TOF, NRT, NT were associated with OHRQoL defined by OHIP-14K after controlling for potential confounders encompassing socio-demographic factors, health behaviors, and general/oral health problems. Second, gender could modify its association. Hence, this cross-sectional study aimed to evaluate the adjusted association of oral health indicators with poor OHRQoL among Korean elders and to investigate its effect modification by gender.

## Methods

### Study design

This cross-sectional study was approved by the Institutional Review Board for Human Subjects at the Seoul National University School of Dentistry (approval number: S-020190017 and C-1803-117-932). The written informed consent was obtained from each participant. Data of this study was from the baseline data of community health education cohort, a collaboration between Medical and Dental Health starting from 2018 in Songbuk-Gu, Seoul, Korea. Participants joined the cohort study voluntarily. Oral health status and systemic health status were assessed by trained dental and medical health professionals, who received calibration training for data collection beforehand.

### Study population

Songbuk-Gu with 0.44 million residents in Seoul metropolitan city with 9.8 million residents in 25 Gus (city level administrative division) was select as a pilot program area of the community health promotiom program for Korean elders by Korea Centers for Disease Control and Prevention (KCDC), because Songbuk-Gu was a representative cluster of elders in Korea [25]. The proportion of population aged 65 and over was 16.5%, which was almost the same as the average of 16.0% in Seoul and in Korea [26]. The participants were randomly recruited in all 20 stratified Dongs (administrative sub-divisions) of Songbuk-Gu. They voluntarily registered to the survey after taking the information about the program by local health center personnel via phone call. On the day of survey, the participants joined the survey at the local government health center for this study.

The inclusion criteria was five-fold: (1) elder aged 65 years and above who lived in Songbuk-Gu, (2) elders who do not live in nursing homes or clinics, (3) people without critical diseases such as cancer and paralysis, (4) people able to communicate and agree to follow the study procedures with self-written informed consent and (5) people joined the oral examination for this study. From total of 73,158 elders aged 65 and above, 743 participants were recruited in this study. Out of them, 68 elders did not join the oral examination. Finally, 675 elder participants who had oral examination were included in the final analysis.

### Assessment of oral health-related quality of life

OHRQoL was measured by the Korean version of the Oral Health Impact Profile (OHIP-14K) [9] by face-to-face interview. For each questionnaire item, participants were asked “How frequently they had experienced the impact of the item during the last 12 months?” and provided the response using a Likert scale (5 points scale), which coded from 0 to 4: 0 = 'never', 1 = 'hardly ever', 2 = 'occasionally', 3 = 'fairly often', 4 = 'very often'. The total score of OHIP-14K, ranged from 0 to56, was calculated by summing up the score of responses of the 14 questionnaire items, the higher scores of which indicated poorer OHRQoL. The prevalence of poor OHRQoL by OHIP-14K was determined by the percentage of adults who reported a negative impact (response codes: 3 'fairly often' and 4 'very often') on one or more of the 14 items. The others who had response codes only from 0 to 2 in all items were considered as fair OHRQoL by OHIP-14K.

### Assessment of oral health indicators

Items of oral health indicators were included: dental status such as dentate and denture, NRT, NT [19, 27]. Additionally, TOF, measuring the maximum force during voluntary maximum clenching, was suggested to be the key determinant of masticatory performance [28], which was proved to have a positive association with OHRQoL [29].

Dental status, NRT, NT were examined by dentists. Dental status was divided into dentate and denture, which included partial and complete dentures. Fixed prosthodontics such as bridges and implants were evaluated as rehabilitated teeth. Root tips and teeth indicated for extraction were considered as missing teeth. Wisdom teeth were excluded from the analysis.

TOF as the maximal occlusal force was evaluated using Dental Prescale II 50H (GC Corp., Tokyo, Japan), a dedicated scanner (GT- X830, EPSON, Tokyo, Japan), and analysis software (Bite Force Analyzer, GC Corp.). TOF was evaluated in Newton (N). This system consists of pressure-sensitive horseshoe-shaped films selected to fit participants' arches, and participants were instructed to bite the film in the intercuspal position as strongly as possible in three seconds. Denture wearers were recommended to keep their dentures in the mouth during the measurement. The analysis was performed after calibration, and dentists carried out manual removal of artifacts according to the manufacturer's instruction. For the reliability of TOF, 10% of the film was planned to retest. The inter-class correlation coefficient between two dentists for 50 films was 0.97 and the intra-class correlation coefficient between two times tests of each dentist for 20 films was 0.96.

According to our unpublished data, elders with TOF lower than 330 N, NRT ≤ 24 and NT ≤ 14 were more likely to have chewing problems, affecting their quality of life. Therefore, in this study, we used 330 N, 24, and 14 as the cut-off value of TOF, NRT, NT, respectively.

### Assessment of confounders

Participants were interviewed in-person to obtain the socio-demo-behavioral confounders, which include factors such as age, gender, education level as social background and alcohol drinking, smoking history as health-related behavioral factors.

Participants were under general health assessment and physical examination performed by physicians, and blood samples were collected at the field survey center in the morning after 8 h of fasting. Metabolic syndrome was defined as having three or more factors among the following factors [30]: (1) Obesity (body mass index (body kg/height m^2^) ≥ 25); (2) Total triglyceride ≥ 150 mg/dL; (3) HDL cholesterol: male < 40 mg/dL, female < 50 mg/dL; (4) Hypertension: systolic blood pressure ≥ 130 mmHg or diastolic blood pressure ≥ 85 mmHg; (5) HbA_1_C ≥ 5.3%. Although the results were within the normal range, diseases were counted if the participant was using medication.

For periodontal examination, clinical attachment loss of all remaining natural teeth excepted 3rd molar was measured by dentists using UNC probe according to the guideline "Staging and grading of periodontitis: Framework and proposal of a new classification and case definition" [31]. Tooth loss due to periodontitis was determined using interview by dentists. Periodontitis was classified into 2 groups: No (healthy or stage I–II) and Yes (stage III–IV).

Frailty was evaluated based on the "FRAIL" scale [32], which consisted of 5 domains scoring ranged from 0 to 5 (one point for each domain), including fatigue, resistance, ambulation, illness, and loss of weight. Less than 3 points indicated no frailty, and 3 points or above was frailty.

### Assessment of effect modification

Effect modification of gender was explored using stratified analysis, because previous studies [33–36] reported the different association of masticatory function and tooth loss with cognitive impairment in gender. Especially, an effect modifier is determined if the stratified association, compared to the non-stratified association, shows significant difference (10% or more) between them [37].

### Statistical analysis

The outcomes were OHRQoL (poor versus fair). The main explanatory variables were oral health indicators: dental status, TOF, NRT, and NT. Age, gender, education level, smoking, drinking, metabolic syndrome, frailty, and periodontitis were considered as confounders.

Differences in characteristics between the prevalence of ‘fair’ and ‘poor’ OHRQoL using OHIP-14K were compared using bivariate analyses such as T‐test and chi‐square test. Demographics and characteristics of the participants were described using mean values with standard deviations for continuous variables and frequency distributions for categorical variables. A chi‐square test was performed to compare differences in categorical variables, and *T*‐test was applied for continuous variables with two groups.

Multiple multivariable logistic regression analysis was applied to evaluate the association (odds ratio, [OR]) of OHRQoL using OHIP-14K (fair versus poor) with main explanatory variables. Confounders were included in the model for adjustment. The stratified analysis by gender was also applied to determine the role of gender in the association. When applied the stratified analysis by gender, confounders excluding gender were contained.

The significance level was set at *p* < 0.05, and all data analyses (Additional file 1) were analyzed using SPSS version 25.0 (SPSS, Inc., Chicago, IL).

## Results

The prevalence of poor OHRQoL was 43.0%, which was higher in women, less-educated elders, alcohol non-drinkers and frailty elders (*p* < 0.05). Age, smoking, periodontitis, metabolic syndrome did not show the significant difference between the poor and fair OHRQoL groups (*p* > 0.05) (Table 1).

**Table 1:** Characteristics of participants according to OHRQoL by OHIP-14K (N = 675)

Variable	N	OHRQoL		*p*-value
		Fair (n = 385)	Poor (n = 290)	
Age, year	675	76.08 ± 5.24	76.16 ± 5.40	0.848
*Gender*				**0.047**
Men	219	137 (62.6)	82 (37.4)	
Women	456	248 (54.4)	208 (45.6)	
*Educational level*				**0.008**
Middle school or less	512	279 (54.5)	233 (45.5)	
High school or more	152	102 (67.1)	50 (32.9)	
Not informed	11	4 (36.7)	7 (63.6)	
*Alcohol drinking*				**0.007**
No	219	112 (51.1)	107 (48.9)	
Yes	438	267 (60.9)	171 (39.1)	
Not informed	18	6 (33.3)	12 (66.7)	
*Smoking*				0.631
No	443	270 (61.0)	208 (39.0)	
Yes	202	119 (55.4)	83 (44.6)	
Not informed	30	15 (50.0)	15 (50.0)	
*Periodontitis*				0.126
No	123	79 (64.2)	44 (35.8)	
Yes	490	268 (54.7)	222 (45.3)	
Not informed	62	38 (61.3)	24 (38.7)	
*Metabolic syndrome*				0.072
No	294	156 (53.1)	138 (46.9)	
Yes	381	229 (60.1)	152 (39.9)	
*Frailty*				**0.011**
No	541	352 (65.1)	218 (34.9)	
Yes	109	48 (44.0)	61 (56.0)	
Not informed	25	14 (56.0)	11 (44.0)	

Values denote as number (raw percentage) for categorical variables and mean ± standard deviation (SD) for continuous variables

*p*-values were obtained from *T*-test for continuous variables and chi-square test for categorical variables

Bold denotes statistical significance at *p* < 0.05

OHRQoL: Fair denotes OHIP-14K < 3: never, hardly ever, occasionally; Poor denotes OHIP-14K ≥ 3: fairly often, very often

Smoking: “No” denotes to never smoked, “Yes” denotes to past and current smoker

Alcohol drinking: “No” denotes to drunken, “Yes” denotes to past and current drinker

Metabolic syndrome: “No” refers to two or less factors, “Yes” refers to three or more factors among five factors: Obesity (body mass index (body kg/height m^2^) ≥ 25); Total triglyceride ≥ 150 mg/dL; HDL cholesterol: Male < 40 mg/dL, Female < 50 mg/dL or medication for dyslipidaemia; Hypertension: systolic blood pressure ≥ 130 mmHg or diastolic blood pressure ≥ 85 mmHg or medication for hypertension; Glycated hemoglobin ≥ 5.3% or medication for diabetes

Periodontitis: followed by guideline “Staging and grading of periodontitis: Framework and proposal of a new classification and case definition” [51] classified into 2 groups: No (healthy or stage I–II) and Yes (stage III–IV)

**Fig. 1:**
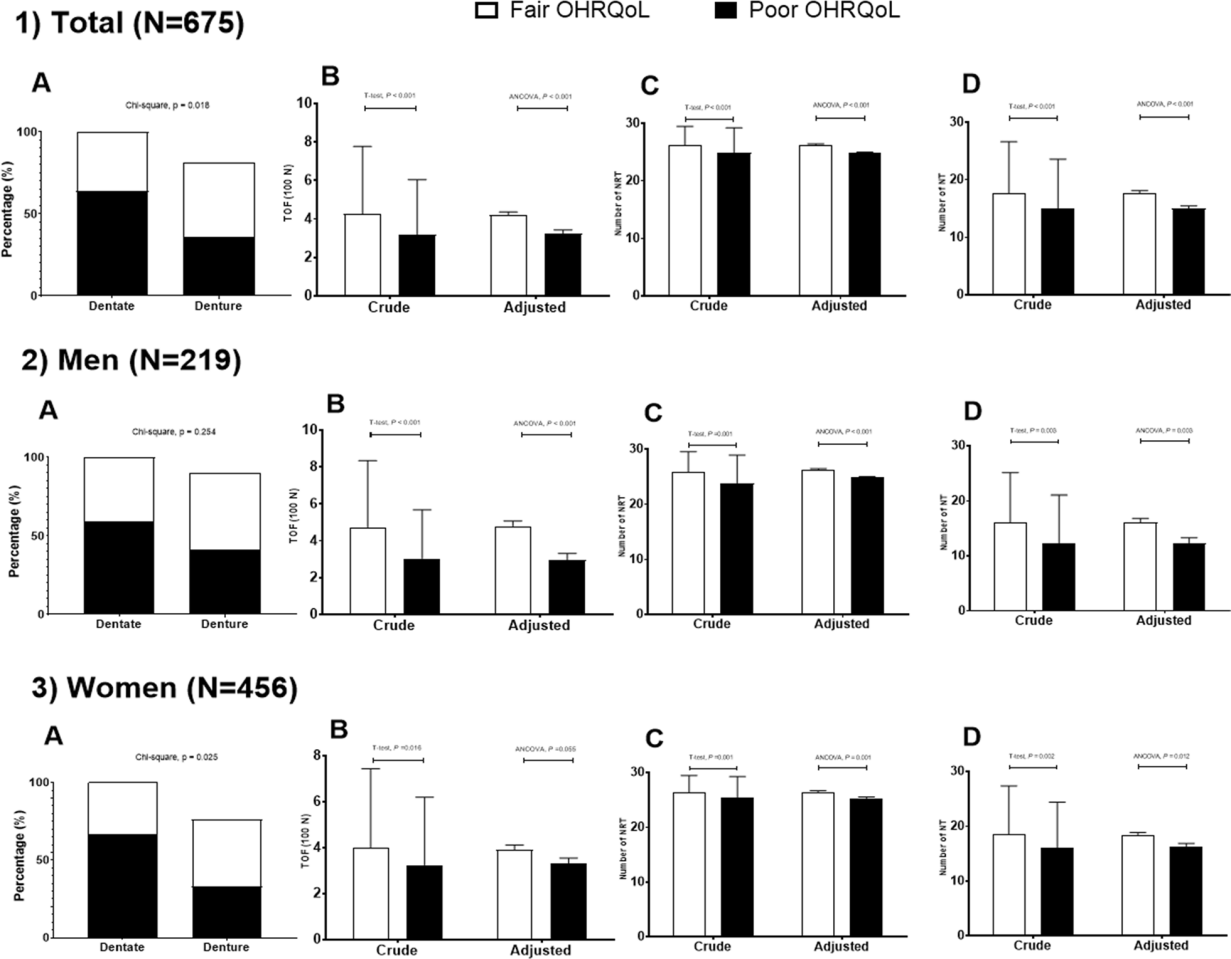
Gender stratified distribution in oral health indicators according to OHRQoL by OHIP-14K (poor versus fair) (n = 675) (**1**) Total; (**2**) Men; (**3**) Women; (**A**) Dental status; (**B**) Total occlusal force (TOF) (unit = 100 N); (**C**) Number of total natural and rehabilitated teeth (NRT); (**D**) Number of natural teeth. Error bar denotes standard deviation for crude value and standard error for adjusted value. Crude values were obtained from the T-test and adjusted values from analysis of covariance (ANCOVA) in a general linear model adjusted for age, gender (only for total sample), educational level, drinking, smoking, periodontitis, metabolic syndrome, and frailty

Regarding to oral health indicators, participants with poor OHRQoL had a lower prevalence of dentate, TOF, NRT, and NT than those with fair OHRQoL in both crude and adjusted analysis (Fig. 1). Among dentate participants, 60.7% of them had fair OHRQoL and 39.3% of them had poor OHRQoL (*p* = 0.02). Participants with poor OHRQoL showed lower TOF (in crude [mean ± standard deviation]: 318 ± 286 N vs 425 ± 352 N; in adjusted [mean ± standard error]: 324 ± 0.2 N vs 420 ± 0.2 N), NRT (in crude: 24.9 ± 4.3 vs 26.2 ± 3.3; in adjusted: 24.8 ± 0.2 vs 26.3 ± 0.2), NT (in crude: 15.0 ± 8.6 vs 17.7 ± 9.0; in adjusted: 15.0 ± 0.5 vs 17.8 ± 0.4) than those with fair OHRQoL (*p* < 0.05).

In terms of the adjusted association of oral health indicators with poor OHRQoL, NRT showed the most strongest impact on poor OHRQoL, followed by TOF and NT (*p* < 0.05) (Table 2). Dental status was not associated with poor OHRQoL. Those with NRT ≤ 24, NT ≤ 14, and TOF < 330 N increased the risk of poor OHRQoL by 2.3 times (OR = 2.26, confidence interval [CI] 1.54–3.31), 1.5 times (OR = 1.45, CI 1.02–2.07), and 1.5 times (OR = 1.47, CI 1.06–2.04), respectively.

**Table 2:** Adjusted association of oral health indicators and confounders with poor OHRQoL by OHIP-14K (n = 675)

Variables	N	Model 1	Model 2	Model 3	Model 4
		OR (95% confidence interval)			
*Dental status*					
Dentate	407	1			
Denture	268	1.25 (0.89–1.76)			
*TOF, Newton*					
≥ 330	293		1		
< 330	382		**1.47 (1.06–2.04)**		
*NTR*					
≥ 25	518			1	
≤ 24	157			**2.26 (1.54–3.31)**	
*NT*					
≥ 15	431				1
≤ 14	244				**1.45 (1.02–2.07)**
Age	675	1.00 (0.96–1.02)	0.99 (0.96–1.02)	0.99 (0.96–1.02)	0.99 (0.96–1.02)
*Gender*					
Men	219	1	1	1	1
Women	456	1.48 (0.93–2.35)	1.47 (0.92–2.34)	1.58 (0.99–2.53)	1.50 (0.95–2.39)
*Education level*					
Middle school or less	512	1	1	1	1
High school or more	152	0.71 (0.48–1.07)	0.72 (0.48–1.07)	0.71 (0.47–1.07)	0.73 (0.48–1.09)
Not informed	11	2.32 (0.66–8.24)	2.25 (0.63–8.01)	2.78 (0.78–9.91)	2.20 (0.62–7.83)
*Alcohol*^†^					
No	219	1	1	1	1
Yes	438	0.72 (0.51–1.02)	0.72 (0.51–1.02)	0.72 (0.51–1.03)	0.73 (0.52–1.04)
Not informed	18	1.55 (0.50–4.85)	1.57 (0.50–4.92)	1.74 (0.55–5.50)	1.47 (0.47–0.64)
Smoking*					
No	443	1	1	1	1
Yes	202	1.20 (0.75–1.93)	1.24 (0.78–1.99)	1.17 (0.73–1.88)	1.15 (0.71–1.86)
Not informed	30	1.29 (0.52–3.19)	1.31 (0.53–3.26)	1.44 (0.58–3.57)	1.32 (0.53–3.27)
*Periodontitis*					
No	123	1	1	1	1
Yes	490	1.46 (0.95–2.27)	1.51 (0.96–2.38)	1.41 (0.91–2.18)	1.40 (0.90–2.18)
Not informed	62	1.19 (0.62–2.30)	1.17 (0.61–2.26)	1.29 (0.67–2.49)	1.14 (0.59–2.19)
*Metabolic syndrome*^‡^					
No	294	1	1	1	1
Yes	381	0.76 (0.55–1.05)	0.76 (0.55–1.05)	0.69 (0.50–0.96)	0.76 (0.55–1.05)
*Frailty*					
No	541	1	1	1	1
Yes	109	**1.71 (1.11–2.64)**	**1.67 (1.09–2.57)**	**1.63 (1.06–2.53)**	**1.71 (1.11–2.63)**
Not informed	25	1.04 (0.45–2.38)	1.09 (0.47–2.52)	1.06 (0.46–2.45)	1.05 (0.46–2.41)

OHRQoL: Fair denotes OHIP-14K < 3: never, hardly ever, occasionally; Poor denotes OHIP-14K ≥ 3: fairly often, very often

TOF, total occlusion force; NRT, number of total natural and rehabilitated; NT, number of remaining natural teeth

Bold denotes statistical significance at *p* < 0.05

Odd ratio: obtained by multivariable logistic regression adjusted for all variables in each model

Periodontitis: followed by guideline “Staging and grading of periodontitis: Framework and proposal of a new classification and case definition” [51] classified into two groups: No (healthy or stage I–II) and Yes (stage III–IV)

^*^Smoking: No denotes never smoked; Yes denotes past and current smoker

^†^Alcohol drinking: “No” denotes “never drunken”; “Yes” denotes “past and current drinker”

^‡^Metabolic syndrome: No denotes two or fewer factors, Yes denotes three or more factors among five factors: obesity (body mass index (body kg/ height m^2^) ≥ 25); Total triglyceride ≥ 150 mg/dL; HDL cholesterol: Male < 40 mg/dL, Female < 50 mg/dL or medication for dyslipidemia; Hypertension: systolic blood pressure ≥ 130 mmHg or diastolic blood pressure ≥ 85 mmHg or medication for hypertension; Glycated hemoglobin ≥ 5.3% or medication for diabetes

Model 1: model of dental status, − 2 Log likelihood = 885.978, Cox&Snell R square = 0.052

Model 2: model of total occlusion force, − 2 Log likelihood = 882.296, Cox&Snell R square = 0.058

Model 3: model of total natural and rehabilitated teeth number, − 2 Log likelihood = 870.134, Cox&Snell R square = 0.074

Model 4: model of natural teeth number, − 2 Log likelihood = 883.342, Cox&Snell R square = 0.056

**Fig. 2:**
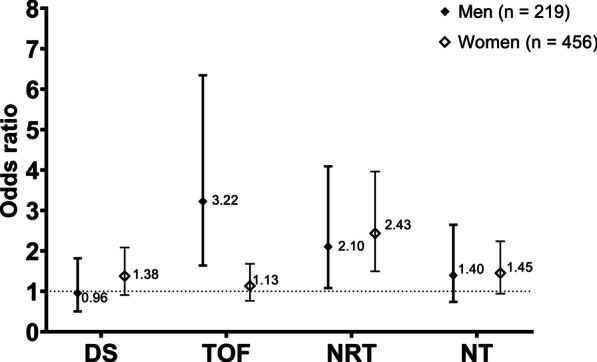
Gender-stratified adjusted association of oral health indicators with poor OHRQoL by OHIP-14K (n = 675). DS: dental status (dentate [reference] versus denture); TOF: Total occlusion force (≥ 330 N [reference] versus < 330 N); NRT: number of total natural and rehabilitated (≥ 25 [reference] versus ≤ 24); NT: number of natural teeth (≥ 15 [reference] versus ≤ 14). Odds ratio (OR) was adjusted for age, education level, smoking, drinking, periodontitis, metabolic syndrome, and frailty in the multivariable logistic regression model. A diamond (black for men, white for women indicates OR, and bars indicate a 95% confidence interval. The horizontal dotted line is the reference as the null of association (OR = 1)

As to the effect modification by gender, its association with poor OHRQoL was highly modified in both men (TOF < 330 N) and women (NRT ≤ 24) (Fig. 2). In men, the association of TOF increased from OR of 1.47 to OR of 3.22 with 95% CI of 1.64–6.34. In women, the association of NRT with poor OHRQoL increased from OR of 2.26 to OR of 2.43 with 95% CI of 1.49–3.96. The stratified analysis induced to make the association of denture and NT ≤ 24 with poor OHRQoL non-signoficant (*p* > 0.05).

## Discussion

Our cross-sectional study showed that oral health indicators such as TOF, NRT and NT were independently associated with poor OHRQoL in the Korean elders after controlling for various confounders. Low TOF, NRT and NT were more likely to have the risk of poor OHRQoL, which supported the previous evidence [11–15]. Interestingly, gender could modify the association. To the best of our knowledge, it is the first evidence on the association between them. The association of low TOF with poor OHRQoL was highly modified in men, while the association of low NRT was modified in women.

The major strengths of this study are five-fold. Firstly, the participants were recruited from the general population, which could decrease the selection bias than participants were recruited from nursing homes or clinics. Secondly, OHRQoL was assessed by OHIP-14K, which showed acceptable reliability and validity with the most widely used OHIP-49 [9]. Thirdly, this study analyzed the data collected directly by medical and dental health professionals during clinical examination not based on self-assessment; hence the classification bias was minimal. Fourthly, stratified analysis was performed to clarify the modification of the association by gender. Finally, the association was controlled with well-known potential confounders, including socio-demographic factors, behavioral factors, periodontitis and common systemic ailments.

Our findings were in line with the previous studies, confirming that NT and NRT were associated with OHRQoL [12–14, 38]. In a birth cohort of 32-year olds in Newzeland, one or more missing teeth could reduce the QoL of the young participants [39]. The presence of at least ten teeth in each jaw was the most important dental condition in dental function to discriminate OHRQoL in the Chinese middle age citizens [40]. The number of teeth is essential to maintain the chewing ability, which in turn, affects the general health, nutritional status, and QoL [41]. Chewing function is also impaired by the decreased occlusal force [42]. Therefore, a lower TOF could be associated with the poor OHRQoL via affecting the mastication and nutritional status. Another oral health indicator that could be associated with OHRQoL is dental status [15, 16]. In the present study, this association was significant only in crude analysis, which was not significant after adjustmnets of confounders and the gender stratification. It was speculated that the quality of the denture may affect the mastication and OHRQoL. Overall, poor oral health indicators were associated with poor OHRQoL. Thus, keeping natural teeth and an adequate oral rehabilitation for the lost teeth could improve occlusal functions, and could positively impact on patients' physical, social, and psychological well-being [43], which could increase the OHRQoL.

In this study, we found that women were more likely to have poor OHRQoL and gender modified the association between the oral health indicators and poor OHRQoL. One possible explanation was that men had a higher tolerance to oral disease and pain than women, thanks to the influence of masculinity on men's well-being [44]. In contrast, women were more unsatisfied with their appearance and showed greater concerns about their oral health, which may raise the poor judgment of their OHRQoL [45]. Indeed, the self-perceived OHRQoL was different between gender by the factors at different stages of the life course [18]. Moreover, under the condition of the traditional Korean culture, rehabilitation of lost teeth in adulthood was more difficult in women than in men. This may explain the association of NRT with poor OHRQoL were modified in women in our study. Meanwhile, that of TOF was modified in men. This may be due to men having a higher TOF and thickness of masseter and temporal muscles than women [46, 47].

This study observed that socio-demo-behavioral factors show complex interrelationships with other variables. Educational level, drinking and frailty had significant differences between the poor and fair impact on OHRQoL in the bivariate relationship, but non of them except frialty were not significant in the multivariable logistic regression model including various confounders. Our data also showed that periodontitis had no association with OHRQoL in the elderly, which supported some previous reports [15, 48], but not supported some other results [24, 49]. It is possible that having had periodontitis over time, the elders could adapt gradually to the discomfort condition, leading to diminishing the impact on the OHRQoL [50]. Differences in diagnosis criteria of periodontitis could be another reason for these controversial results.

Despite its strengths, some limitations were available in this study. Firstly, this is a cross-sectional study; therefore, it could not evaluate the causal relationship between the oral health indicators and poor OHRQoL. Secondly, other factors such as masticatory muscle function were not considered, which could affect the variation of occlusal force measurement among elders with similar oral conditions. Finally, the quality of denture was not assessed meticulously, which could affect the satisfaction chewing ability. Regardless of these limitations, our study was appropriate enough to evaluate the association between the oral health indicators and OHRQoL.

## Conclusion

Oral health indicators consisting of total occlusal force, number of total natural and rehabilitated teeth and number of natural teeth were independently associated with OHRQoL using OHIP-14K among Korean elders after controlling for various confounders. Moreover, gender modified the association. Hence, oral health practitioners should be aware that preventive and/or curative management for keeping natural teeth and the rehabilitation of missing teeth to recover the occlusal force may be essential for reducing poor OHRQoL.

## Supplementary Information

**Additional file 1**. Data file used for this study.

## Data Availability

All data generated or analysed during this study are included in this published article [and its Additional file 1].
