# Correction: Gender modified association of oral health indicators with oral health-related quality of life among Korean elders

**DOI:** 10.1186/s12903-022-02241-y

**Published:** 2022-05-26

**Authors:** Huong Vu, Phuc Thi-Duy Vo, Hyun-Duck Kim

**Affiliations:** 1 Department of Preventive and Social Dentistry, School of Dentistry, Seoul National University, 101 Daehak-Ro, Jongno-Gu, Seoul, 03080 Republic of Korea; 2 Department of Immunology and Molecular Microbiology, School of Dentistry and Dental Research Institute, Seoul National University, Seoul, Republic of Korea; 3 Dental Research Institute, Seoul National University, Seoul, Republic of Korea

## Correction to: BMC Oral Health (2022) 22:168 10.1186/s12903-022-02104-6

In this article, the bars of “Denture” in Fig. 1. 1A, 2A, and 3A were not correctly scaled. They should be presented as 100%. The corrected figure is given below.

**Fig. 1:**
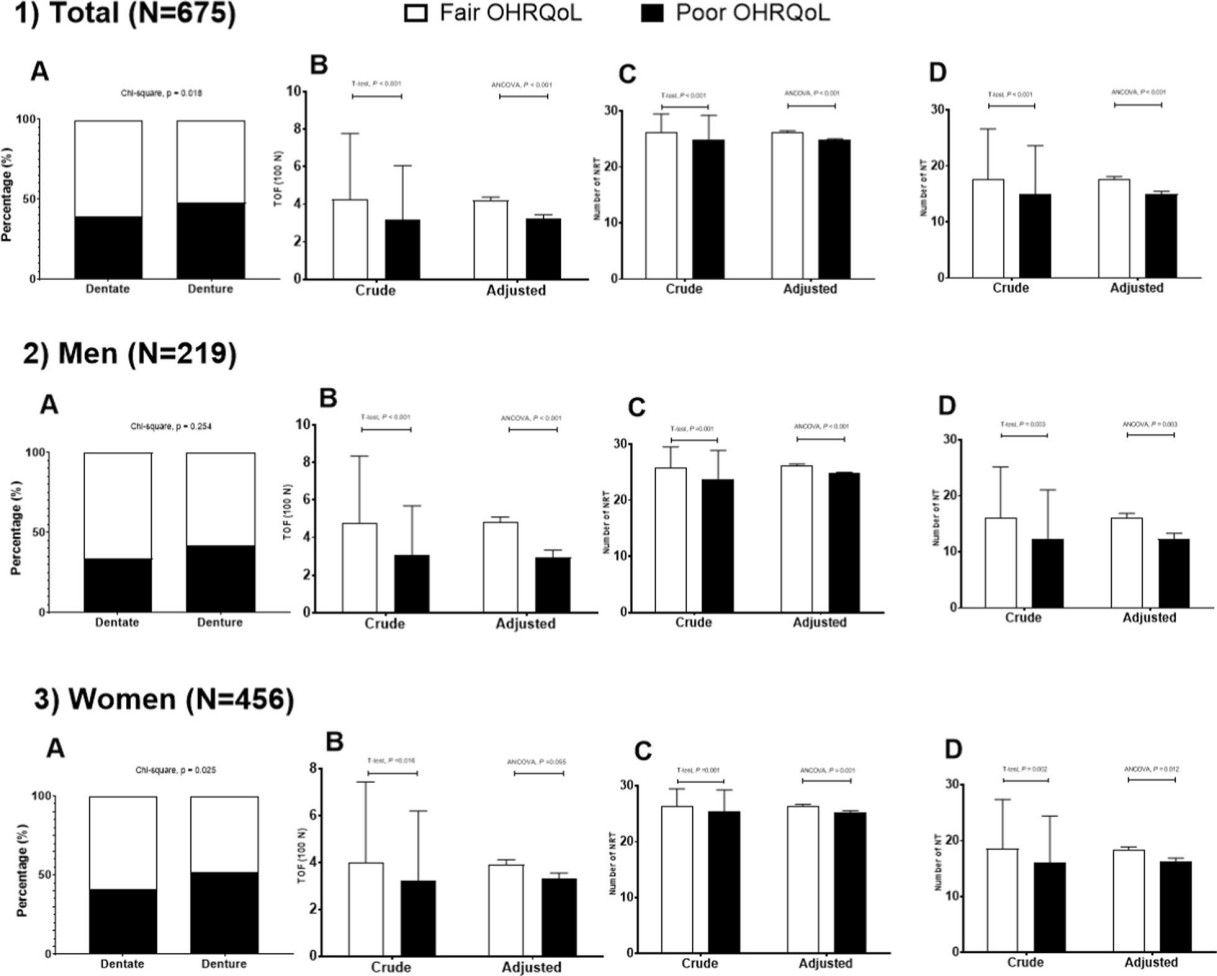
Gender stratified distribution in oral health indicators according to OHRQoL by OHIP-14K (poor versus fair) (n = 675) (**1**) Total; (**2**) Men; (**3**) Women; (**A**) Dental status; (**B**) Total occlusal force (TOF) (unit = 100 N); (**C**) Number of total natural and rehabilitated teeth (NRT); (**D**) Number of natural teeth. Error bar denotes standard deviation for crude value and standard error for adjusted value. Crude values were obtained from the T-test and adjusted values from analysis of covariance (ANCOVA) in a general linear model adjusted for age, gender (only for total sample), educational level, drinking, smoking, periodontitis, metabolic syndrome, and frailty

